# Charting a course to sustainability

**DOI:** 10.1093/af/vfab035

**Published:** 2021-09-06

**Authors:** Kristen A Johnson

**Affiliations:** Department of Animal Sciences, Washington State University, Pullman, WA, USA

“Sustainability” is not an obtainable target; rather, it is a framework for decision-making over time. Thus, it is appropriate to periodically examine the status of livestock production systems across the world as these systems change within the sustainability framework. *Animal Frontiers* has published several issues in which components of animal agriculture are examined in the context of a sustainable food system (Table 1). The diversity of themes represented in the issues listed in Table 1 is large, which illustrates the complexity of animal production systems. This issue, “Charting a Course to Sustainability,” is an examination of the current state of the science needed to create livestock production systems that assist in the creation of a sustainable system.

**Table 1.** Articles on sustainable food production published in *Animal Frontiers*

Year	Title	Citation
2011	Fork to Farm: The carbon footprint.	2011, 1(1):3–15.
2011	International trade and livestock production: future prospects for the beef industry.	2011, 1(2):6–59.
2012	Reducing the impact of animal production on the water supply: increasing knowledge is the only solution	2012, 2(2):1–49.
2015	Land-use challenges for animal agriculture	2015, 5(4):4–42.
2016	The use of performance enhancing technologies in global livestock production	2016, 6(4):4–55.
2019	Climate change: impact on livestock and how we can adapt.	2019, 9(1):3–76.
2019	Foods of animal origin: a prescription for global health.	2019, 9(4): 3–57.
2020	The future of animal protein: feeding a hungry world.	2020, 10(4):5–72.
2021	More than meat: contributions of livestock systems beyond meat production.	2021, 11(2):3–71.
2021	Charting a course to sustainability	2021, 11(3):5–103.

At its most basic level, a sustainable system comprises three pillars: economic, environmental, and social (Brundtland Commission, 1987). Over time, other disciplines have deepened the definition, and a new multidisciplinary entity, sustainability science, has developed. Moore et al. (2017) created a definition in the context of health care and identified five components that are also relevant to animal agriculture. These components include a time function, delivery of implementation strategies, occurrence and maintenance of system change, behavioral adaptation or evolution, and maintenance of system benefits. All of these components have been found in livestock production systems across the world and are a part of the FAO Sustainable Food Systems concept and framework (http://www.fao.org/3/ca2079en/CA2079EN.pdf; FAO, 2018). A recent synthesis of the sustainability literature has identified six capabilities that are essential to frameworks through which sustainable systems can be created (Clark and Harley, 2020). These include a mechanism by which sustainable development can be recognized (an indicator), promotion of equity, adaptability to disruption, creation of an environment in which sustainable development can occur, linkage of new knowledge to change, and arrangements to allow people to work together. Critical to the development of a sustainable system is a need for tools, including system models, that can be used to query whole systems as a part of decision-making (Ahmed and Sundaram, 2007). To create these tools, models need sustainability indicators, sufficient information, and a modeling framework that can integrate societal, economic, and environmental factors.

This issue highlights much of the work that continues across the world. Casey (2021) profiles the history, progress, and challenges in South Africa as that country strives for sustainable livestock production systems. The authors also highlight the policy framework that governs the production and the need for supportive policies. Policy options are the topic of the paper by Haugen-Kozyra (2021). This paper describes many of the market-based tools that are available in Canada to assist in creating sustainable production systems. Canada has been a proving ground for innovative solutions including the identification of best management practices and the creation of a Certified Sustainable Beef Framework.

Significant progress has been made in pastoral systems in New Zealand, Ireland, and Costa Rica. Much has been accomplished in New Zealand (NZ) meat production systems over 30 yr, and Moot and Davidson (2021) discuss that progress and identify new challenges toward sustainable meat production. Much of the emphasis and progress in the creation of sustainable systems have focused around farm-level management changes associated with the education and dedication of the NZ meat industry to embrace sustainable practices. Ireland has also had success in its journey to sustainable livestock production (O′Mara et al., 2021) and has ambitious plans to move forward to more effectively link soil, air, and water resources and education to livestock production systems. This is a similar approach to that described by Montengro and Abarca (2021) in Costa Rica. Costa Rican livestock systems have benefited from policy initiatives such as the National Strategy for Low Carbon Livestock (MAG-MINAE, 2015) that support livestock systems.

As mentioned previously, part of creating sustainable ruminant livestock systems is the ability to develop indicators of sustainability, including the measurement of greenhouse gases and other emissions. Balehegn et al. (2021) update the status and challenges that exist broadly across the continent of Africa. Many challenges are associated with extreme climates and smallholder farms, and many of the solutions will benefit agriculture across the world as climate changes. Several of the technologies identified by Balehegn et al. (2021) are also described by Dillon et al. (2021) who discusses the state of livestock sustainability in the United States. One of the major points that Dillon et al. (2021) raises is the role of models in the development of sustainable decision-making frameworks and the need for those models to have adequate data to make them useful and robust.

In summary, significant progress has been made in the identification of indicators and the creation of an environment in which sustainable development can occur. The SARS-COV-2 pandemic has identified barriers to adaptability to sudden changes that can and are being addressed. New knowledge is available as the field progresses with the development of models and decision tools and that knowledge is actively translated to livestock producers through active outreach and extension programs. There are also increasing numbers of arenas in which people can work together such as the Global Roundtable for Sustainable Beef and Dairy Sustainability Framework. This issue of *Animal Frontiers* demonstrates not only the importance of the task to create sustainable animal agricultural production systems but also the progress we have made.

## About the Authors

**Kristen Johnson** is a Professor and Scientist in Animal Sciences at Washington State University, Pullman, WA. She conducts research in beef cattle energetics and has applied this work to examine issues to improve the efficiency and environmental sustainability of ruminant production systems. She also teaches undergraduate and graduate classes and mentors graduate students in animal sciences.
